# Analysis of Cadmium Contamination in Lettuce (*Lactuca sativa* L.) Using Visible-Near Infrared Reflectance Spectroscopy

**DOI:** 10.3390/s23239562

**Published:** 2023-12-01

**Authors:** Lina Zhou, Leijinyu Zhou, Hongbo Wu, Lijuan Kong, Jinsheng Li, Jianlei Qiao, Limei Chen

**Affiliations:** 1 College of Engineering and Technology, Jilin Agricultural University, Changchun 130118, China; zhoulina976430@163.com (L.Z.); zhouleijinyu8514@163.com (L.Z.); whb05082023@163.com (H.W.); konglijuan630@sina.com (L.K.); jinshengl@jlau.edu.cn (J.L.); 2 College of Horticulture, Jilin Agricultural University, Changchun 130118, China; qiaojianlei918@163.com

**Keywords:** spectral parameters, cadmium, inversion model, regression analysis, SPAD value

## Abstract

In order to rapidly and accurately monitor cadmium contamination in lettuce and understand the growth conditions of lettuce under cadmium pollution, lettuce is used as the test material. Under different concentrations of cadmium stress and at different growth stages, relative chlorophyll content of lettuce leaves, the cadmium content in the leaves, and the visible-near infrared reflectance spectra are detected and analyzed. An inversion model of the cadmium content and relative chlorophyll content in the lettuce leaves is established. The results indicate that cadmium concentrations of 1 mg/kg and 5 mg/kg promote relative chlorophyll content, while concentrations of 10 mg/kg and 20 mg/kg inhibit relative chlorophyll content. The cadmium content in the leaves increases with increasing cadmium concentrations. Cadmium stress caused a “blue shift” in the red edge position only during the mature period, while the red valley position underwent a “blue shift” during the seedling and growth periods and a “red shift” during the mature period. The green peak position exhibited a “blue shift”. After model validation, it was found that the model constructed using the ratio of red edge area to yellow edge area and the normalized values of red edge area and yellow edge area effectively estimated the cadmium content in lettuce leaves. The model established using the normalized vegetation index of the red edge and the ratio of the peak green value to red shoulder amplitude can effectively estimate the relative chlorophyll content in lettuce leaves. This study demonstrates that the visible-near infrared spectroscopy technique holds great potential for monitoring cadmium contamination and estimating chlorophyll content in lettuce.

## 1. Introduction

In China, approximately 20% of cultivated land is affected by heavy metal pollution [1]. Among this land, the rate of excessive cadmium pollution is 7%, making it the primary pollutant in contaminated soil areas [2]. Cadmium is characterized by its high toxicity, strong mobility, and resistance to degradation. It is easily absorbed and accumulated by plants, and it can enter the human body through the food chain, posing a threat to human health [3]. Therefore, rapid and accurate monitoring or identification of heavy metal stress levels in plants is of significant importance for ensuring food safety.

Currently, monitoring of plant heavy metal pollution is predominantly carried out using chemical and spectroscopic techniques. Among them, chemical methods offer high accuracy but are complex, destructive, and not suitable for rapid and large-scale monitoring, requiring significant human and material resources [4]. Spectral techniques can detect the levels of heavy metal pollution in plants and assess their growth conditions by analyzing changes in the reflectance spectra of plant leaves. This allows for rapid and non-destructive monitoring of heavy metal contamination in plants [5].

Many researchers have conducted in-depth studies on the monitoring of heavy metal pollution in plants and the inversion of chlorophyll content using the variation characteristics of plant spectra. Li et al. [6] analyzed the hyperspectral response characteristics of chicory leaves under cadmium stress and established a monitoring model for the cadmium mass ratio in chicory leaves using first-order derivative spectroscopy–partial least squares regression (FDR-PLS). They believed that in situ hyperspectral technology could achieve rapid and accurate monitoring of cadmium pollution. Abdel-Rahman et al. [7] used hyperspectral data and partial least squares (PLS) to establish a quantitative monitoring model for cadmium content in Swiss chard leaves, achieving good prediction results. Chen et al. [8] analyzed the hyperspectral characteristics of tobacco leaves under cadmium stress. The results showed that the ratio vegetation index (RVI) and normalized difference vegetation index (*NDVI*) in the spectral indices had a significant correlation with cadmium content in tobacco leaves and had a good predictive effect on the cadmium content in tobacco leaves. Liu et al. [9] established a model for the inversion of chlorophyll content in soybean leaves using spectral indices. The spectral indices difference index (DI) and first-derivative difference index (FDDI) showed the highest correlation with chlorophyll content, and the resulting model can provide a reference for large-scale monitoring of soybean growth conditions. Wang et al. [10] used spectral characteristics to invert the chlorophyll content in Sabina vulgaris leaves. The vegetation indices (*NDVI*) and vegetation cover index (m*NDVI*) showed a high correlation with the chlorophyll content, and the inversion model established had a high accuracy.

In order to achieve rapid and accurate monitoring of cadmium pollution in lettuce and understand the growth status of lettuce under cadmium contamination, this study conducted detection and analysis of visible-near infrared reflectance spectra, cadmium content, and SPAD values in lettuce leaves under cadmium pollution. A model for the inversion of cadmium content and chlorophyll content in lettuce leaves under cadmium pollution was constructed. The purpose is to provide a theoretical basis and reference for the safe production and quality control of lettuce.

## 2. Materials and Methods

### 2.1. Materials and Experimental Design

The experiments were conducted in May 2023 within the campus of Jilin Agricultural University in Changchun, Jilin Province (125°42′ E, 43°82′ N). The pot planting method was employed, using lettuce (*Lactuca sativa* L. cv. Grand Rapids) as the test material, purchased from Kuishou Agricultural Technology Co., Ltd. (Langfang, China). The experimental soil used was uncontaminated nutrient soil with an organic matter content of 12.59 g/kg, total nitrogen content of 0.727 g/kg, available phosphorus content of 0.007 g/kg, and available potassium content of 0.15 g/kg. The soil was sieved with a screen to remove impurities and separate it into fine particles. Then, the soil was allowed to stand in a dry, ventilated area for 3 days. The cadmium content in the experimental soil was set at 5 concentration gradients: 0 (control group, CK), 1, 5, 10, and 20 mg/kg. Distilled water was used as the solvent, and cadmium nitrate was used as the external source of cadmium for addition. A 200 mL solution of each concentration of cadmium was prepared. The different concentrations of cadmium solution were sprayed layer-by-layer onto the corresponding experimental soil and thoroughly mixed by turning over the soil. After aging for 10 days, the soil was transferred into pots with dimensions of 480 cm × 230 cm × 160 cm, with 1.5 kg of soil being added to each pot [11,12]. Three replicates were set for each treatment level. When the lettuce seedlings reached the stage of two leaves and one heart, uniformly growing and healthy seedlings were selected and transplanted into the pots. Three seedlings were planted in each pot. To ensure the normal growth of the lettuce, an adequate water supply was provided throughout the experiment. The positions of the pots were changed every two days throughout the entire experimental period to ensure even exposure to light.

### 2.2. Spectral Data Acquisition

The visible-near infrared reflectance spectra data of lettuce leaves were measured at three different time points: 15 days (seedling stage) after cadmium stress, 30 days (growth stage) after cadmium stress, and 45 days (maturity stage) after cadmium stress. The measurements were conducted for leaves from the five treatment groups. The spectrometer selected for this study was the AvaSpec-ULS2048 multi-purpose fiber optic spectrometer, manufactured by Aventes in the Netherlands. This spectrometer has a wavelength range of 200–1100 nm and a spectral resolution of 0.05–20 nm. The light source used was the AvaLight-DHc full-range compact light source, also produced by Aventes. The deuterium lamp covers the wavelength range of 200–400 nm, while the tungsten halogen lamp covers the wavelength range of 400–2500 nm.

The measurements were conducted under the following weather conditions: clear sky, no wind, and few clouds. The measurements were taken between 10:00 AM and 2:00 PM. During data collection, the first step was to connect the fiber optic probes separately to the spectrometer and light source. Then, the reflection probe was secured using a reflection probe holder in such a way that the angle between the reflection probe and the leaf surface was 45°. Finally, employing a deuterium–halogen lamp as the light source that was allowed to preheat for 8 min, white balance calibration and measurements were performed. White balance calibration was conducted every 30 min throughout the entire experimental process. For sample selection, the top 2 or top 3 leaves were chosen for measurement. During the measurement, the main veins of the leaves were avoided. Eight spectra data were collected for each lettuce leaf, and any abnormal spectra data were removed. Finally, the average value of the remaining spectra data was taken as the reflection spectrum data for that particular lettuce leaf. Under each cadmium stress treatment at different growth stages, three spectral curves were obtained, resulting in a total of 45 curves.

### 2.3. SPAD Value Determination

The SPAD values of the leaves were determined using a handheld chlorophyll meter (SPAD-502Plus, Konica Minolta, Chiyodaku, Japan). The top 2 or top 3 leaves were selected for measurement while avoiding the main leaf veins. Each leaf was measured three times, and the average of these measurements was taken as the SPAD value for that particular leaf.

### 2.4. Leaf Cadmium Content Determination

The measurement of leaf cadmium content was conducted using a digestion method. Firstly, the corresponding leaves for spectral data collection were washed, dried, and ashed. Then, a Touchwin 2.0 microwave digestion instrument was used for digestion. The specific steps were as follows: weighing 0.15 g of the sample into a 30 mL polytetrafluoroethylene crucible, adding 8 mL of a mixed acid solution (concentrated nitric acid:hydrofluoric acid:perchloric acid = 3:3:1), heating with an open lid at 250 °C until the perchloric acid volatilized (the sample was completely dried at this point), and then turning off the power. When the temperature dropped to approximately 180 °C, 8 mL of aqua regia and 10 mL of internal standard mixed solution were added to the crucible, mixed well, filtered, and set aside. Finally, 250 µL of the digestion solution was taken, mixed with 5 mL of 3% nitric acid solution, and measured for cadmium content in the leaves using an inductively coupled plasma–mass spectrometry (ICP-MS) instrument (300D, Perkinelmer, Waltham, MA, USA).

### 2.5. Data Processing and Analysis

#### 2.5.1. Data Processing Methods

The AvaSoft 8.5 spectral acquisition software was used for collecting spectral data. The collected spectral data were preprocessed and subjected to correlation analysis using Origin 2021 software. SPSS 23 software was employed for conducting significance analyses and regression analyses on the data.

The Pearson correlation coefficient method was employed to assess the correlation between the leaf cadmium content, relative chlorophyll content (SPAD value), and spectral parameters. Strongly correlated parameters were identified, with R-values closer to 1 or −1 indicating stronger correlations, while values closer to 0 indicated weaker correlations.

To determine if there are significant differences in the mean SPAD values among different groups, a significance test is used. Typically, the significance levels of 5% and 1% are employed as criteria. If the significance level is less than 5% or 1%, the difference is considered to be significant or highly significant, respectively.

The regression model can be utilized in various fields such as prediction, association analysis, and causal inference. Therefore, in this study, we chose to employ a regression model to investigate the relationship between the leaf cadmium content, SPAD value, and spectral characteristic parameters. The goodness of fit of the model for inferring leaf cadmium content from SPAD values is assessed based on the F-value and R-squared (R^2^). A larger F-value indicates a better fit of the model, reflecting a stronger explanatory power of the model for the dependent variable. The closer R^2^ is to 1, the better the regression model fits the observed values, indicating a closer relationship between the two variables.

#### 2.5.2. Calculation Method for Spectral Characteristic Parameters

In this study, spectral characteristic parameters such as the green peak, red valley, red edge, red edge amplitude, and normalized difference vegetation index at 705 nm (NDVI_705_) were used for analysis. The specific calculation methods are shown in Table 1.

## 3. Results and Discussion

### 3.1. Effects of Cadmium Stress on the Biochemical Parameters of Lettuce

#### 3.1.1. Effects of Cadmium Stress on the SPAD Value of Lettuce

**Figure 1:**
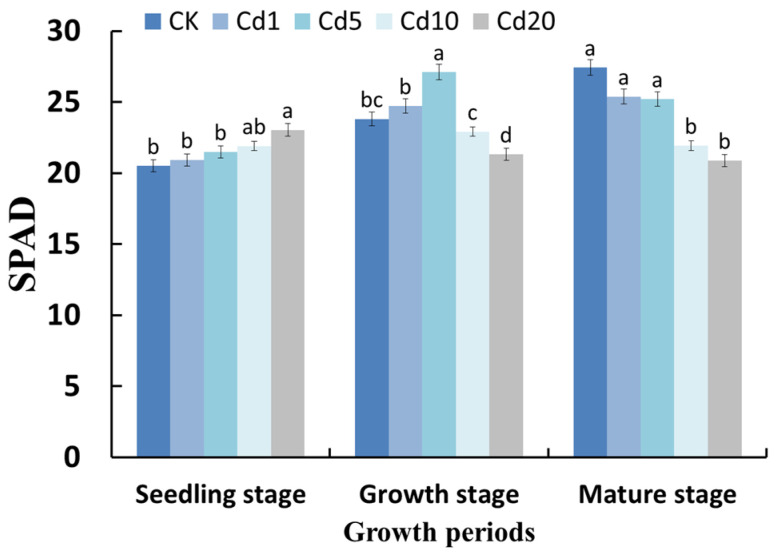
Effects of cadmium stress on the SPAD values of lettuce. Note: Different lowercase letters indicate significant differences at the *p* < 0.05 level.

Figure 1 shows the changes in SPAD values in lettuce leaves at different growth stages under cadmium stress. During the seedling stage, the SPAD values increased with increasing cadmium concentrations in the soil. During the growth stage, the SPAD values increased in the 1 mg/kg and 5 mg/kg cadmium stress groups compared to the control group (CK) but decreased in the 10 mg/kg and 20 mg/kg cadmium stress groups compared to the control group. During the mature stage, the SPAD values of the lettuce leaves gradually decreased with increasing cadmium concentrations in the soil. This study used cadmium nitrate as an exogenous source of cadmium. It is worth noting that nitrate ions can promote the accumulation of chlorophyll in lettuce leaves [13]. However, it should also be noted that nitrate ion and cadmium ion concentrations vary in direct proportion, and under the stress of 10 mg/kg and 20 mg/kg cadmium nitrate, there is an inhibitory effect on the SPAD values. Moreover, similar to the research results obtained by Jia et al. [14,15,16], who used cadmium chloride as an exogenous source of cadmium, the stress of 1 mg/kg and 5 mg/kg cadmium nitrate also exhibited a promoting effect on the SPAD values. Therefore, it can be inferred that under cadmium nitrate stress, cadmium plays a dominant role in affecting the SPAD values. The stress of 1 mg/kg and 5 mg/kg cadmium promotes the SPAD values, possibly due to the ability of metal ions to enhance the metabolism of cytokinin enzymes, thereby promoting cell growth and increasing chlorophyll content. On the other hand, the stress of 10 mg/kg and 20 mg/kg cadmium inhibits the SPAD values, likely because cadmium stress can cause damage to the chloroplast structure in plant leaf cells, leading to hindered biosynthesis of chlorophyll [15,16,17].

#### 3.1.2. Effects of Cadmium Stress on Cadmium Content in Lettuce Leaves

**Figure 2:**
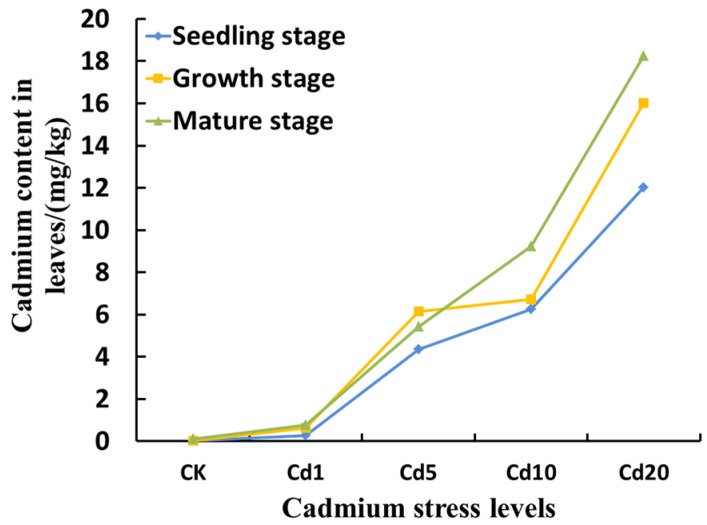
Effects of cadmium stress on the cadmium content in lettuce leaves.

According to Figure 2, the cadmium content in the lettuce leaves at different growth stages showed an increasing trend with the increasing cadmium concentrations in the soil. Lettuce is sensitive to cadmium stress and can effectively accumulate cadmium. The enrichment effect becomes more pronounced with increasing cadmium concentrations. This is attributed to the presence of substances such as organic acids, cellulose, and pectin in the plant cell wall, which can chelate cadmium. As the cadmium concentration increases, the content of cadmium in the cell organelles increases and accumulates continuously [18,19].

### 3.2. Analysis of Spectral Response Characteristics of Lettuce Leaves under Cadmium Stress

#### 3.2.1. Differential Analysis of Visible-Near Infrared Spectra

**Figure 3:**
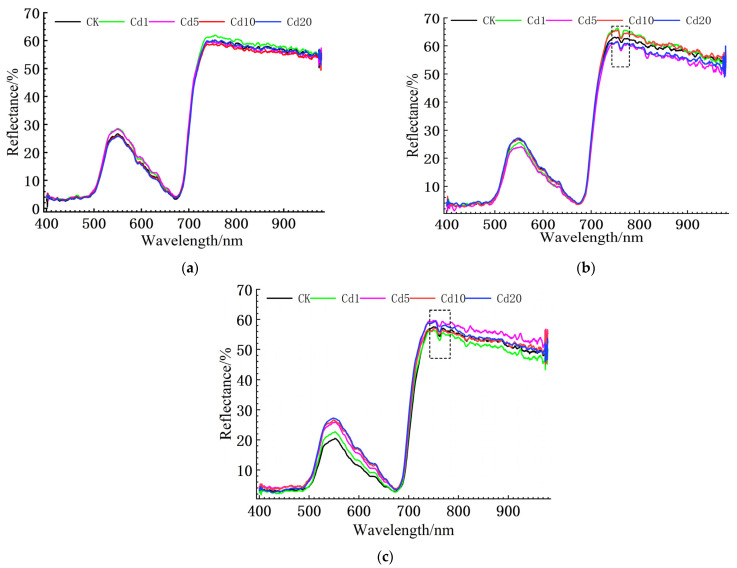
Spectral characteristics of reflectance in lettuce leaves under cadmium stress: (**a**) spectral characteristics of reflectance in lettuce leaf seedlings under cadmium stress; (**b**) spectral characteristics of reflectance in lettuce leaves during the growth stage under cadmium stress; (**c**) spectral characteristics of reflectance in lettuce leaves during the maturation stage under cadmium stress.

Vegetation is sensitive to stress in the visible-near infrared band, and spectral bands beyond 1000 nm are greatly affected by water vapor absorption. Therefore, this paper takes the spectral range of 400–980 nm as the analysis object [20,21]. The utilization of the SG smoothing technique facilitates a more continuous and gradual transition between data points while still retaining the trend and features of the original curve. This approach proves advantageous for subsequent analysis [22]. Therefore, the raw spectral data collected from the five treatments were subjected to SG smoothing, with the parameters set to a second-degree polynomial and 11 smoothing points [23], resulting in the visible-near infrared reflectance spectra curves of the lettuce leaves at different growth stages under cadmium stress shown in Figure 3.

The visible-near infrared reflectance spectra of the lettuce leaves under different cadmium concentrations and growth stages exhibit a high degree of similarity in their characteristic changes. At approximately 670 nm, a distinct absorption valley, known as the “red valley,” is observed, while a reflection peak, known as the “green peak,” appears close to 550 nm. In the wavelength range of 680 nm–750 nm, the reflectance of the lettuce leaves increases sharply, showing a typical “red edge effect”. During the growth and maturation stages, a small absorption valley is observed at approximately 760 nm in the lettuce leaves, which could be attributed to the narrow water absorption band in that wavelength range [24,25].

#### 3.2.2. Analysis of Spectral Characteristic Parameters in Lettuce Leaves

Based on the analysis of the differences in the visible-near infrared reflectance spectra, representative spectral features such as the red edge, red valley, and green peak were selected for further analysis. As shown in Table 2, compared to the CK group, the red edge position showed no change during the seedling stage and growth stage but exhibited a 9 nm “blue shift” during the mature stage. The red valley position initially experienced a “blue shift” followed by a “red shift” (6 nm blue shift during the seedling stage, 7 nm blue shift during the growth stage, and 5 nm red shift during the mature stage). The green peak position underwent a “blue shift” (3 nm blue shift during the seedling stage, 1 nm blue shift during the growth stage, and 3 nm blue shift during the mature stage). When plants are subjected to heavy metal stress, the activity of enzymes required for chlorophyll formation within the plant is inhibited, hindering chlorophyll formation. This leads to an increase in lutein and a decrease in chlorophyll, resulting in a “blue shift” in the spectral features of the red edge and red valley [26,27]. The “red shift” in the red valley position and the “blue shift” in the green peak position during the maturation stage may be related to self-regulation or changes in the leaf structure of lettuce plants under cadmium stress [28,29]. Therefore, the red edge, red valley, and green peak can be used to discriminate the extent of cadmium pollution in lettuce plants.

#### 3.2.3. Analysis of Normalized Difference Vegetation Index (*NDVI*_705_) at the Red Edge

**Figure 4:**
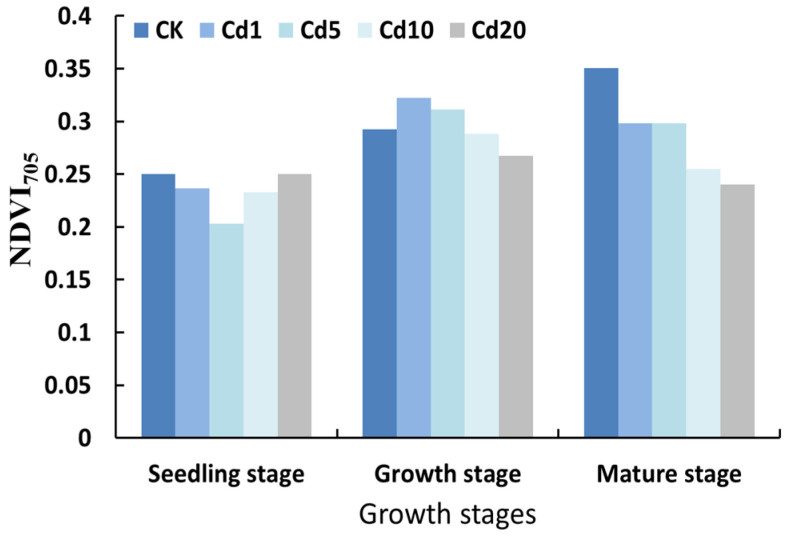
Normalized difference vegetation index (*NDVI*_705_) of lettuce under different cadmium concentrations at different growth stages.

The normalized difference vegetation index (*NDVI*_705_) at the red edge is highly sensitive to the environment and is one of the commonly used indicators for detecting plant stress. Its value ranges from −1 to 1, with a typical range of 0.2 to 0.9 for green vegetation areas. It is generally considered that when the *NDVI*_705_ value is less than 0.2, it indicates that the plant is under stress [27,30]. In this study, the *NDVI*_705_ decreased first and then increased with increasing cadmium concentrations during the seedling stage, but its value was lower than that of the CK group. During the growth stage, the *NDVI*_705_ first increased and then decreased with increasing of cadmium concentrations. During the maturation stage, the *NDVI*_705_ decreased with increasing cadmium concentrations (Figure 4). Under cadmium stress conditions of 10 mg/kg and 20 mg/kg, the *NDVI*_705_ values at the different growth stages were lower than those of the CK group, indicating that the cadmium stress of 10 mg/kg and 20 mg/kg had an impact on the *NDVI*_705_ values of the lettuce and exerted a stress effect on the growth of the lettuce [31].

### 3.3. Correlation Analysis between Spectral Characteristic Parameters, Leaf Cadmium Content, and SPAD Value

**Figure 5:**
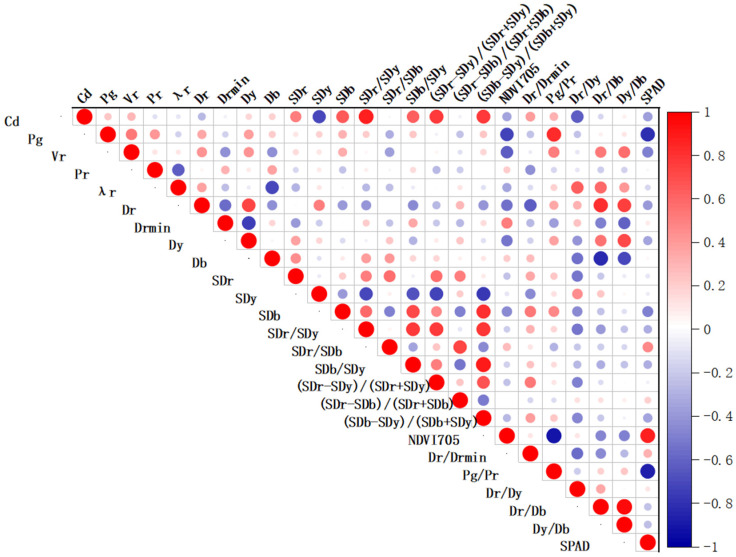
Correlation analysis between spectral characteristic parameters, leaf cadmium content, and SPAD value under cadmium stress.

To screen for sensitive spectral characteristic parameters of lettuce under cadmium stress, a correlation analysis was conducted between 23 spectral characteristic parameters, the leaf cadmium content, and the SPAD value. The results are depicted in Figure 5, where red indicates a positive correlation between two parameters and blue represents a negative correlation. The size of the circles represents the degree of association, with larger circles indicating higher correlations. Among the 23 characteristic parameters, SDy, SDb, SDr/SDy, SDb/SDy, (SDr − SDy)/(SDr + SDy), (SDb − SDy)/(SDb + SDy), and Dr/Dy showed high correlations with leaf cadmium content. The research findings indicate that the spectral characteristic parameters of the “three-edge” region in lettuce leaves are sensitive to cadmium stress. The accumulation of cadmium in plant leaves can be reflected in their physiology through various physiological indicators, such as chlorophyll and cell structure [17]. Slight changes in these indicators can cause alterations in the spectral characteristics of the leaves. In addition, the spectral characteristics of the red edge and yellow edge regions can also serve as diagnostic indicators for cadmium pollution in lettuce [32]. The spectral characteristic parameters of the “three-edge” region are effective wavelengths for monitoring the extent of heavy metal Pb^2+^ pollution, while the blue edge and red edge are effective wavelengths for monitoring the extent of heavy metal Cu^2+^ pollution [33]. Therefore, the spectral characteristic parameters associated with chlorophyll and cell structure can serve as indicators for diagnosing cadmium pollution in lettuce.

Pg, *NDVI*_705_, and Pg/Pr showed high correlations with the SPAD value. Using ratio and normalization methods to construct spectral characteristic parameters has shown better results in estimating leaf chlorophyll content [34]. *NDVI* is effective in reflecting crop growth and eliminating partial radiation errors, making it suitable for dynamic vegetation monitoring [35]. Pg can serve as an important spectral parameter for estimating the SPAD value of tomato leaves under disease stress [36]. The screened spectral characteristic parameters in this study all reached a significant level of correlation with the SPAD value, demonstrating statistical significance. They can be regarded as important spectral characteristic parameters for accurately estimating the chlorophyll content in lettuce leaves under cadmium stress.

### 3.4. Establishment of a Leaf Cadmium Content Inversion Model for Lettuce under Cadmium Stress

According to the results of the correlation analysis, using 45 collected spectral data points as the modeling samples, a regression analysis was conducted using lettuce leaf cadmium content as the dependent variable and spectral characteristic parameters SDy, SDb, SDr/Sdy, SDb/Sdy, (SDr − Sdy)/(SDr + Sdy), (SDb − Sdy)/(SDb + Sdy), Dr/Dy as independent variables. Linear, quadratic, cubic, and logarithmic functions were fitted to the model. The results are presented in Table 3. The cadmium content in the lettuce leaves showed a high correlation with SDr/SDy, SDb/SDy, and (SDr − SDy)/(SDr + SDy), with fitting coefficients (R^2^) of 0.872, 0.781, and 0.792, respectively.

SDr/SDy, SDb/SDy, and (SDr − SDy)/(SDr + SDy) demonstrated better performance in estimating cadmium content in lettuce leaves. However, a study by Gu et al. [32] suggests that the red edge area SDr has the best performance in estimating cadmium content in Chinese cabbage leaves. Chen et al. [8] found that the normalized vegetation index *NDVI* is effective in estimating cadmium content in tobacco leaves. Zhong et al. [37] concluded that *NDVI* has the best performance in estimating cadmium content in various organs of rice. The reason for this difference may lie in the variation of the tested materials. It suggests that different plant species may have different spectral characteristic parameters that are sensitive to cadmium.

### 3.5. Model for SPAD Value Inversion in Lettuce Leaves under Cadmium Stress

The selected spectral parameters, including Pg, *NDVI*_705_, and Pg/Pr, were combined with the lettuce SPAD values for analysis. Linear, quadratic, and cubic regression models were used to fit the SPAD value models. The results are presented in Table 4, where the fitting coefficients (R^2^) between the lettuce leaf SPAD values and Pg, *NDVI*_705_, and Pg/Pr were 0.677, 0.789, and 0.755, respectively.

By constructing spectral indices, redundant spectral information can be effectively removed, thereby improving the accuracy of estimating plant chlorophyll content [38]. In this study, the model for estimating SPAD values using *NDVI*_705_ as the independent variable showed the best fitting effect. This is consistent with the findings of Guo et al. [39] regarding the estimation of corn SPAD values using hyperspectral data, where the original spectral *NDVI* was identified as the optimal parameter for estimating corn SPAD values. However, this is in contrast to the findings of He et al. [34], who reported that the normalized vegetation index had an R^2^ value of 0 when used for quantitative estimation of chlorophyll content in leaves of karst plants. This difference may arise from the variations in environmental conditions and plant species. Therefore, it is necessary to select spectral parameters for estimating SPAD values based on the environmental conditions and plant species.

### 3.6. Model Validation

**Figure 6:**
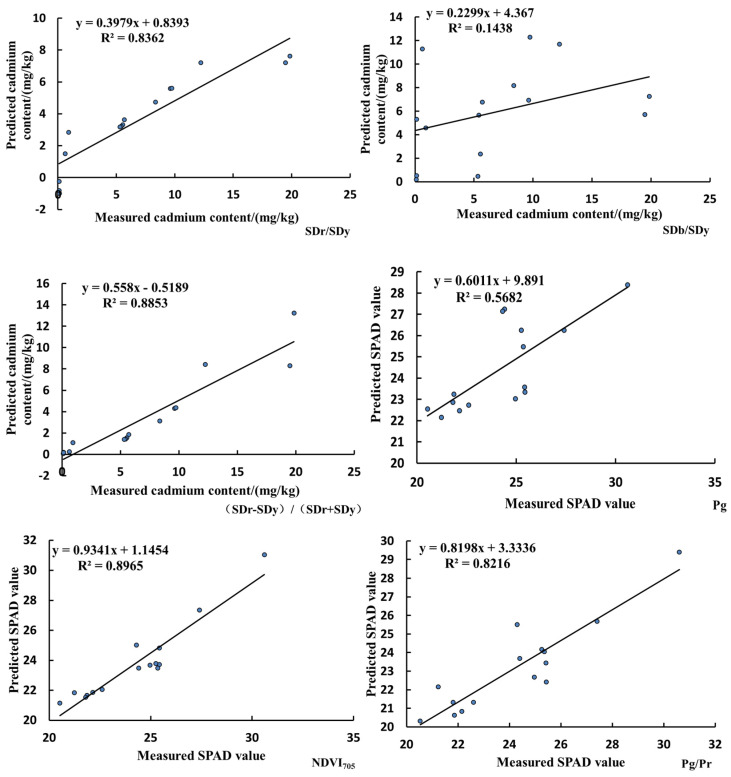
Fitted curve of predicted values and actual measurements.

The spectral data for five samples each in the seedling stage, growth stage, and mature stage were randomly selected to calculate SDr/SDy, SDb/SDy, (SDr − SDy)/(SDr + SDy), Pg, *NDVI*_705_, and Pg/Pr as six spectral characteristic parameters. SDr/SDy, SDb/SDy, and (SDr − SDy)/(SDr + SDy) were used as independent variables to predict the cadmium content in the lettuce leaves, while Pg, *NDVI*_705_, and Pg/Pr were used as independent variables to predict the SPAD value of the lettuce leaves. The predicted values were compared with the actual values, and the results are shown in Figure 6. The SDr/SDy and (SDr − Sdy)/(SDr + Sdy) models can accurately estimate cadmium content in lettuce leaves. The *NDVI*_705_ and Pg/Pr models can accurately estimate the SPAD values of lettuce leaves.

## 4. Conclusions

Lettuce was used as the research object, and a pot planting method with exogenous addition of cadmium was used to study the lettuce leaf SPAD values, cadmium content in the leaves, and visible-near-infrared reflectance spectra under cadmium stress. The following conclusions were drawn:

(1) Under cadmium stress, the SPAD values of the lettuce leaves exhibited inhibition at high concentrations and promotion at low concentrations. Moreover, the cadmium concentration in the lettuce leaves increased with increasing soil cadmium concentrations.
(2) The red edge, red valley, and green peak positions in the reflectance spectra of the lettuce leaves were sensitive to cadmium stress. Under cadmium stress, these positions shifted, and they could be used for preliminary diagnosis of cadmium pollution in lettuce. The normalized difference vegetation index of the red edge (*NDVI*_705_) was lower than the CK group under 10 mg/kg and 20 mg/kg cadmium stress, indicating that lettuce growth was affected by the cadmium stress.
(3) The models corresponding to SDr/SDy and (SDr − SDy)/(SDr + SDy) can effectively estimate the cadmium content in lettuce leaves. The models corresponding to *NDVI*_705_ and Pg/Pr can accurately estimate the SPAD values of lettuce leaves.

This research demonstrates that the utilization of visible-near infrared spectroscopy technology can provide a theoretical basis and reference for the safe production and quality control of lettuce.

## Figures and Tables

**Table 1:** Calculation methods for spectral characteristic parameters.

Feature Parameter	Parameter Description	Wavelength/nm
Pg (Peak green value)	Maximum value of leaf reflectance spectrum	500~600
Vr (Depth of red valley)	Minimum value of leaf reflectance spectrum	600~720
Pr (Red shoulder amplitude)	Maximum value of leaf reflectance spectrum	750~950
Λr (Red edge position)	Wavelength corresponding to the maximum value of the first-order derivative of the leaf reflectance spectrum	670~780
Dr (Amplitude of red edge)	Maximum value of the first-order derivative of the leaf reflectance spectrum	670~780
Drmin (Minimum amplitude of red edge)	Minimum value of the first-order derivative of the leaf reflectance spectrum	670~780
Dy (Amplitude of yellow edge)	Maximum value of the first-order derivative of the leaf reflectance spectrum	560~640
Db (Amplitude of blue edge)	Maximum value of the first-order derivative of the leaf reflectance spectrum	490~530
SDr (Red edge area)	Sum of the first-order derivative of the leaf reflectance spectrum	670~780
SDy (Yellow edge area)	Sum of the first-order derivative of the leaf reflectance spectrum	560~640
SDb (Blue edge area)	Sum of the first-order derivative of the leaf reflectance spectrum	490~530
SDr/SDy	Red edge area/yellow edge area	/
SDr/SDb	Red edge area/blue edge area	/
SDb/SDy	Blue edge area/yellow edge area	/
(SDr − SDy)/(SDr + SDy)	Normalized value of red edge area and yellow edge area	/
(SDr − SDb)/(SDr + SDb)	Normalized value of red edge area and blue edge area	/
(SDb − SDy)/(SDb + SDy)	Normalized value of blue edge area and yellow edge area	/
*NDVI*_705_ (Normalized vegetation index of the red edge)	$NDVI_{705}=(R_{750}-R_{705})/(R_{750}+R_{705})$	*R*_750_ and *R*_705_ represent the spectral reflectance values at 750 nm and 705 nm, respectively
Dr/Drmin	Amplitude of red edge/minimum amplitude of red edge	/
Pg/Pr	Peak green value/red shoulder amplitude	/
Dr/Dy	Amplitude of red edge/amplitude of yellow edge	/
Dr/Db	Amplitude of red edge/amplitude of blue edge	/
Dy/Db	Amplitude of yellow edge/amplitude of blue edge	/

**Table 2:** Spectral characteristic parameters of lettuce leaves at different growth stages under different cadmium concentrations.

Cadmium Treatment	Seedling Stage			Growth Stage			Mature Stage		
	Red Edge Position/nm	Green Peak Position/nm	Red Valley Position/nm	Red Edge Position/nm	Green Peak Position/nm	Red Valley Position/nm	Red Edge Position/nm	Green Peak Position/nm	Red Valley Position/nm
CK	702	551	676	693	548	677	702	550	671
Cd1	702	551	676	693	548	678	702	550	671
Cd5	702	548	673	693	548	676	702	551	671
Cd10	702	551	670	693	548	670	693	551	676
Cd20	702	551	670	693	547	676	702	547	676

**Table 3:** Inversion models for cadmium content in lettuce leaves under cadmium contamination stress.

Spectral Characteristic Parameters	Fitting Model	R^2^	Significance	F
SDy	$y=12.305+0.053x-0.033x^{2}+0.001x^{3}$	0.535	*	4.223
SDb	$y=1.265+0.286x+0.011x^{2}$	0.431	*	4.545
SDr/SDy	$y=-3.934+3.256x-0.305x^{2}+0.009x^{3}$	0.872	**	24.959
SDb/SDy	$y=-2.007+19.988x-8.462x^{2}+0.973x^{3}$	0.781	**	13.041
(SDr − SDy)/(SDr + SDy)	$y=0.186-0.599x-6.829x^{2}+26.942x^{3}$	0.792	**	13.996
(SDb − SDy)/(SDb + SDy)	$y=10.545+15.075x-7.488x^{2}-14.209x^{3}$	0.65	**	6.800
Dr/Dy	$y=-32.046+47.732x-13.561x^{2}$	0.463	*	5.178

Note: * and ** indicate significant correlations at the 0.05 and 0.01 levels, respectively.

**Table 4:** Inversion models for SPAD values in lettuce leaves under cadmium contamination.

Spectral Characteristic Parameters	Fitting Model	R^2^	Significance	F
Pg	$y=21.548+1.043x-0.036x^{2}$	0.677	**	12.569
*NDVI*_705_	$y=29.196-94.046x+259.359x^{2}$	0.789	**	22.407
Pg/Pr	y=47.787−56.575x	0.755	**	40.009

Note: ** indicate significant correlation at the 0.01 levels.

## Data Availability

The data that support the findings of this study are available from the authors upon reasonable request.
